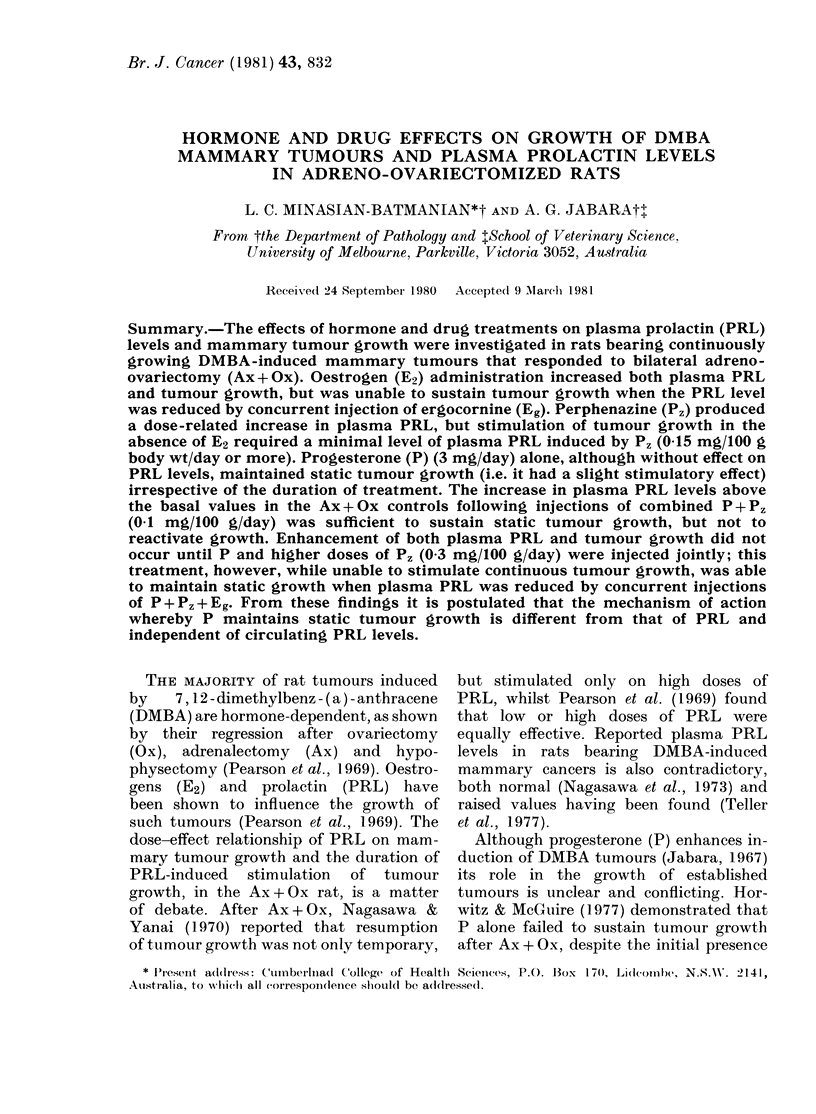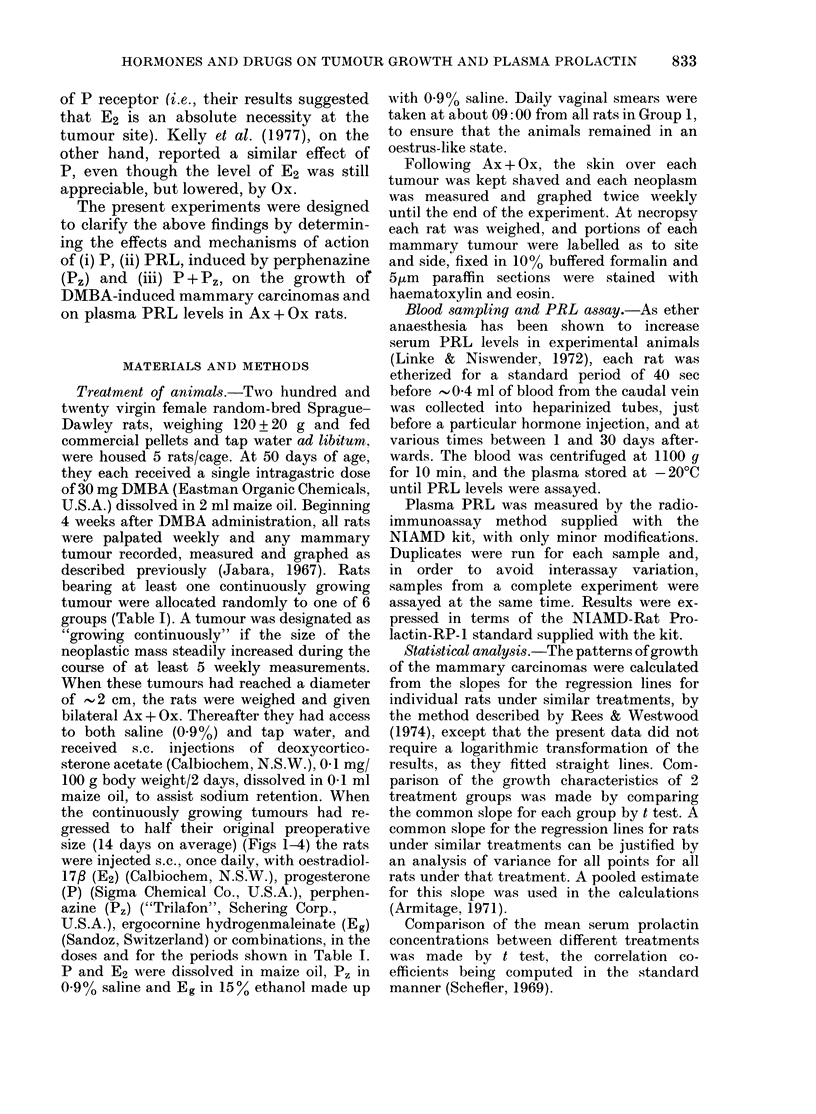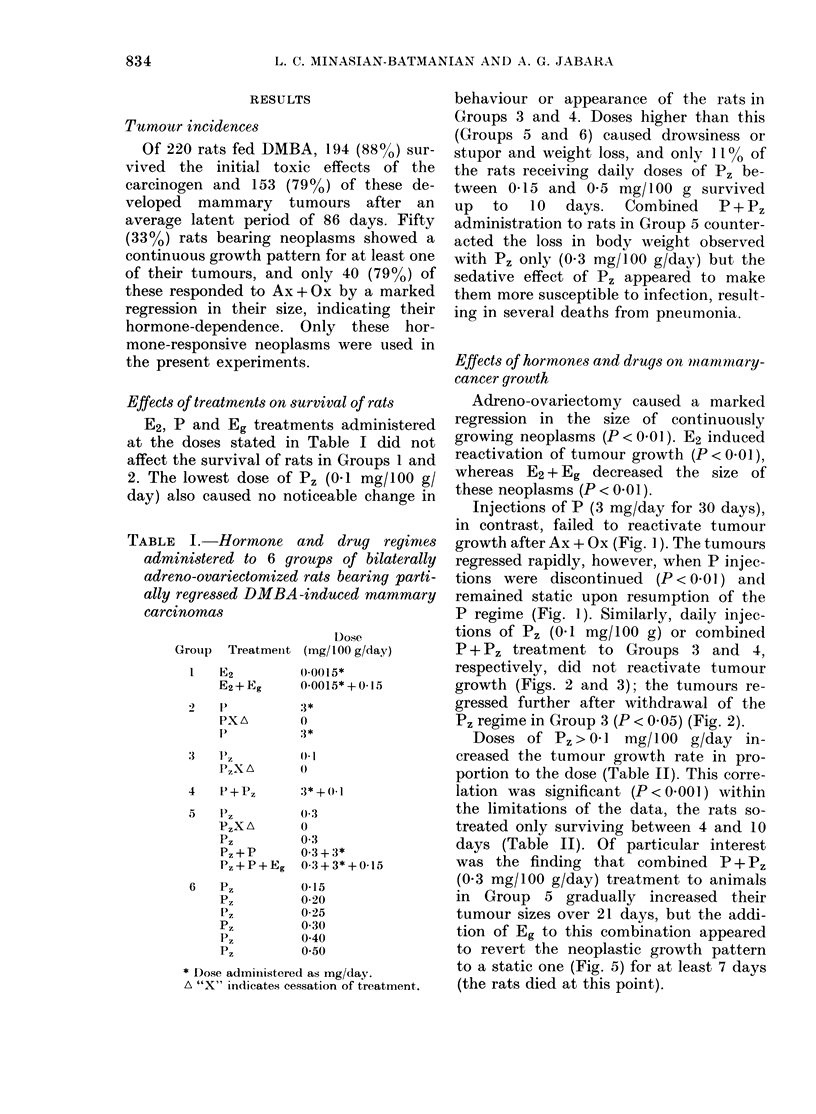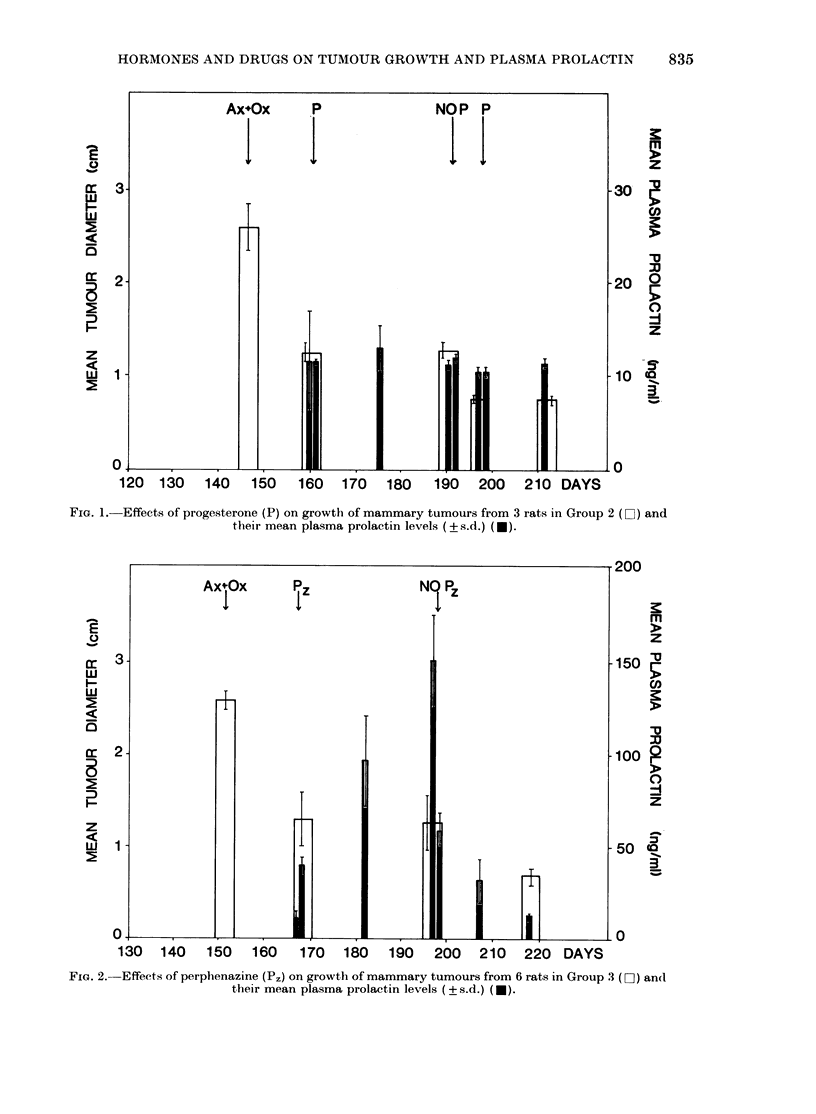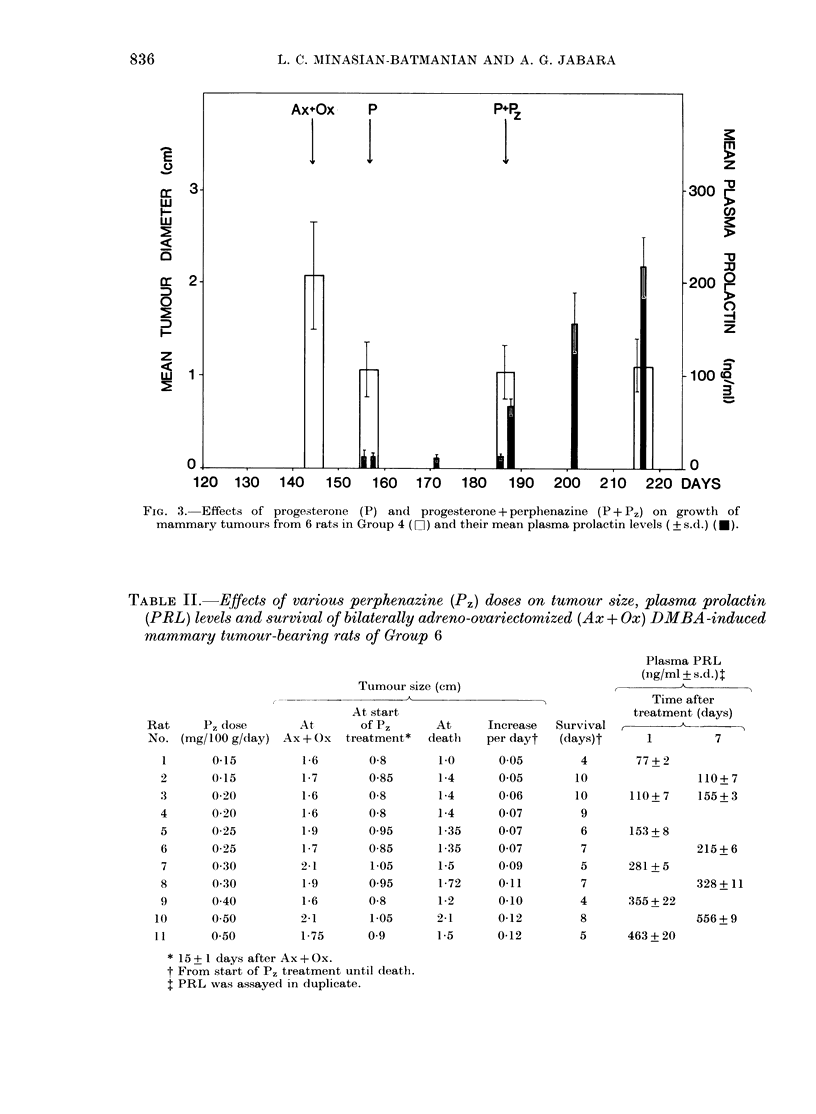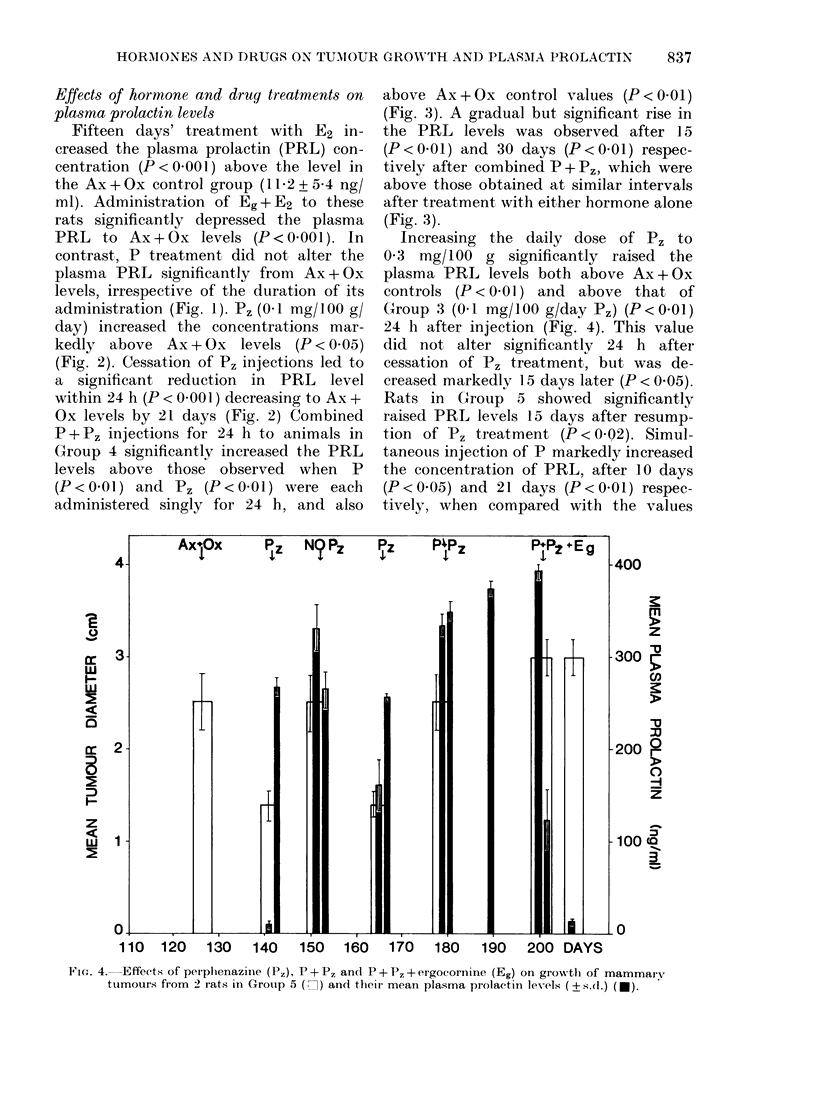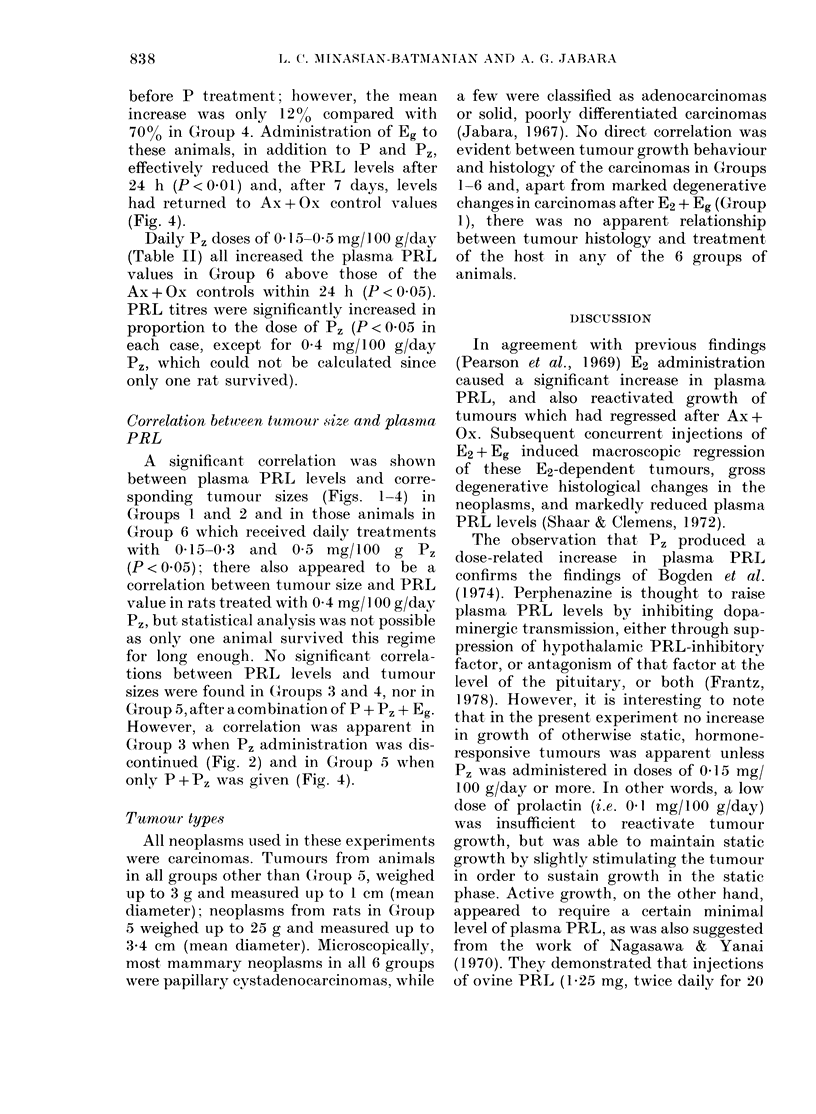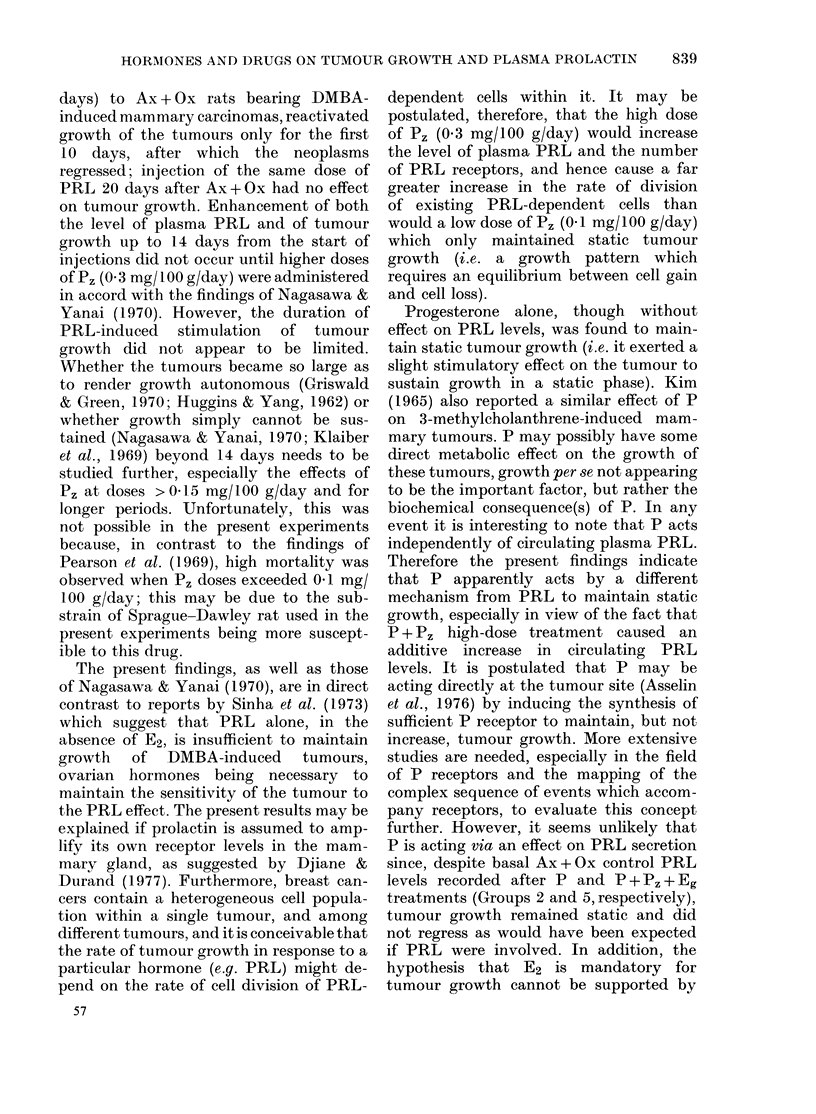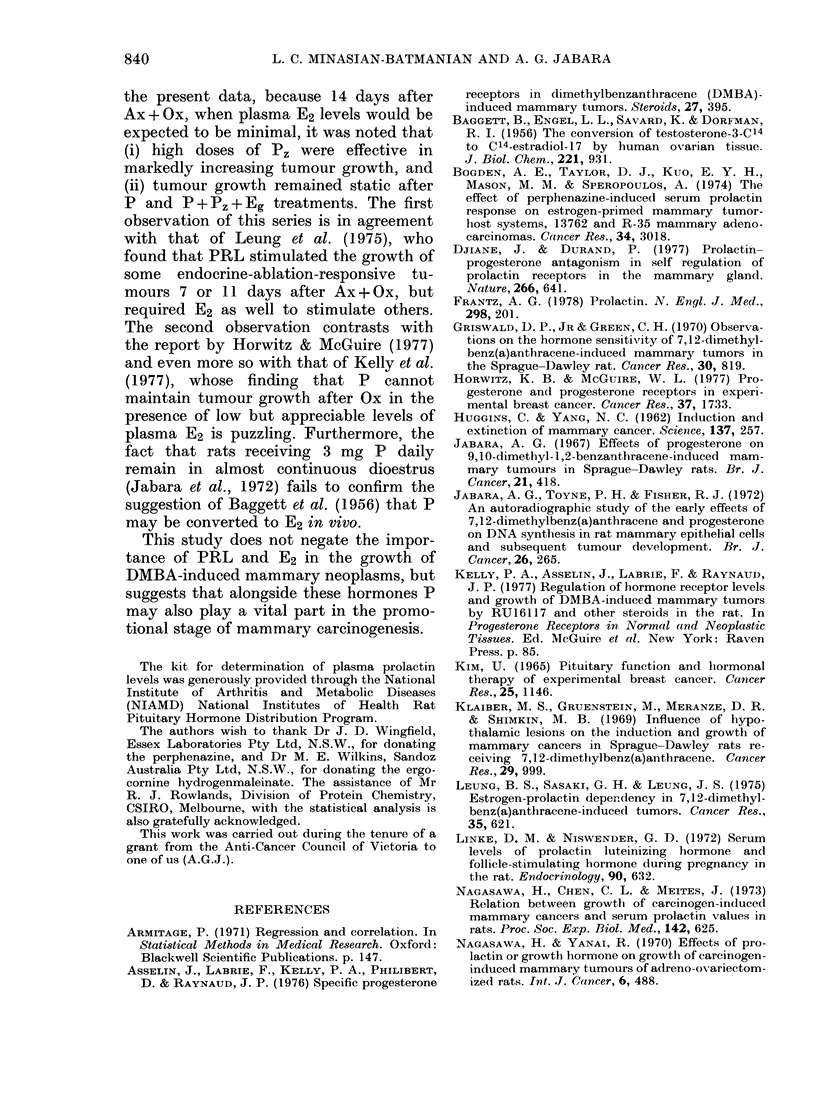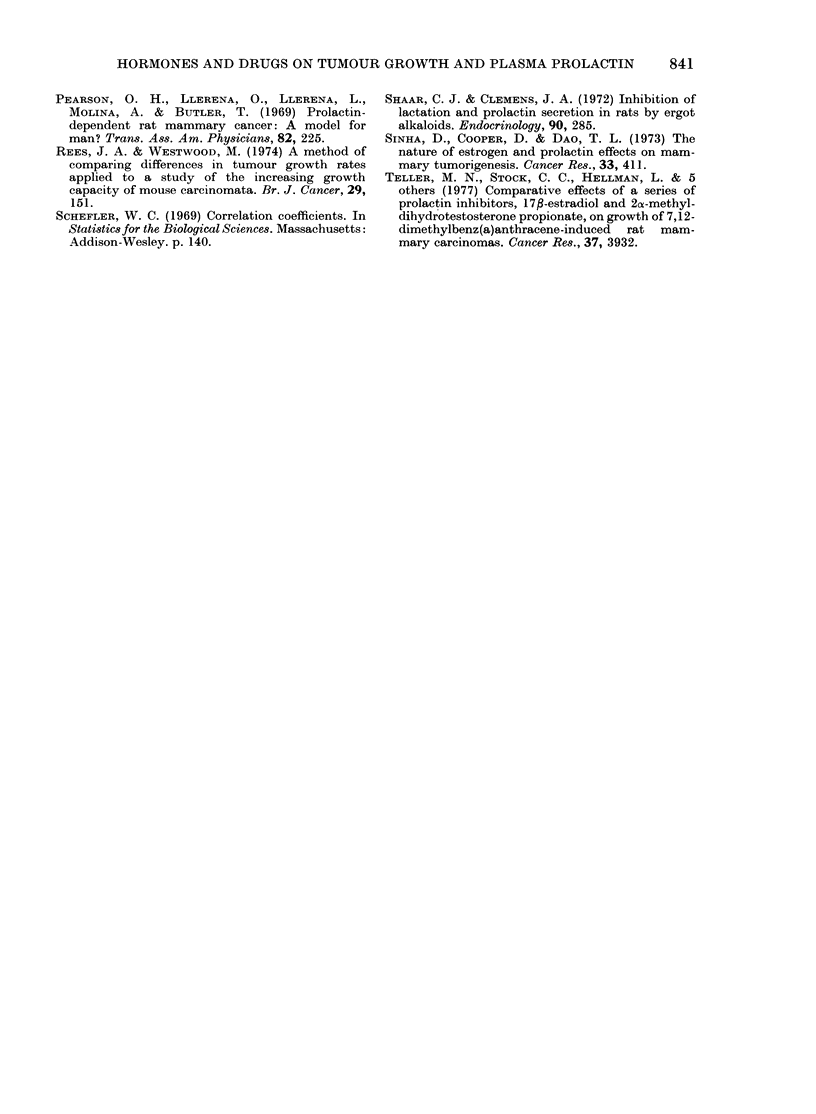# Hormone and drug effects on growth of DMBA mammary tumours and plasma prolactin levels in adreno-ovariectomized rats.

**DOI:** 10.1038/bjc.1981.122

**Published:** 1981-06

**Authors:** L. C. Minasian-Batmanian, A. G. Jabara

## Abstract

The effects of hormone and drug treatments on plasma prolactin (PRL) levels and mammary tumour growth were investigated in rats bearing continuously growing DMBA-induced mammary tumours that responded to bilateral adreno-ovariectomy (Ax + Ox), Oestrogen (E2) administration increased both plasma PRL and tumour growth, but was unable to sustain tumour growth when the PRL level was reduced by concurrent injection of ergocornine (Eg). Perphenazine (Pz) produced a dose-related increase in plasma PRL, but stimulation of tumour growth in the absence of E2 required a minimal level of plasma PRL induced by Pz (0.15 mg/100 g body wt/day or more). Progesterone (P) (3 mg/day) alone, although without effect on PRL levels, maintained static tumour growth (i.e. it had a slight stimulatory effect) irrespective of the duration of treatment. The increase in plasma PRL levels above the basal values in the Ax + Ox controls following injections of combined P + Pz (0.1 mg/100 g/day) was sufficient to sustain static tumour growth, but not to reactivate growth. Enhancement of both plasma PRL and tumour growth did not occur until P and higher doses of Pz (0.3 mg/100 g/day) were injected jointly; this treatment, however, while unable to stimulate continuous tumour growth, was able to maintain static growth when plasma PRL was reduced by concurrent injections of P + Pz + Eg. From these findings it is postulated that the mechanism of action whereby P maintains static tumour growth is different from that of PRL and independent of circulating PRL levels.


Br. J. Cancer (1981) 43, 832

### HORMONE AND DRUG EFFECTS ON GROWTH OF DMBA MAMMARY TUMOURS AND PLASMA PROLACTIN LEVELS IN ADRENO-OVARIECTOMIZED RATS

L. C. MINASIAN-BATMANIAN*† AND A. G. JABARA†‡

*From †the Department of Pathology and ‡School of Veterinary Science, University of Melbourne, Parkville, Victoria 3052, Australia*

Received 24 September 1980 Accepted 9 March 1981

**Summary.—The effects of hormone and drug treatments on plasma prolactin (PRL) levels and mammary tumour growth were investigated in rats bearing continuously growing DMBA-induced mammary tumours that responded to bilateral adreno-ovariectomy (Ax+Ox). Oestrogen ($E_2$) administration increased both plasma PRL and tumour growth, but was unable to sustain tumour growth when the PRL level was reduced by concurrent injection of ergocornine ($E_g$). Perphenazine ($P_z$) produced a dose-related increase in plasma PRL, but stimulation of tumour growth in the absence of $E_2$ required a minimal level of plasma PRL induced by $P_z$ (0·15 mg/100 g body wt/day or more). Progesterone (P) (3 mg/day) alone, although without effect on PRL levels, maintained static tumour growth (i.e. it had a slight stimulatory effect) irrespective of the duration of treatment. The increase in plasma PRL levels above the basal values in the Ax+Ox controls following injections of combined $P+P_z$ (0·1 mg/100 g/day) was sufficient to sustain static tumour growth, but not to reactivate growth. Enhancement of both plasma PRL and tumour growth did not occur until P and higher doses of $P_z$ (0·3 mg/100 g/day) were injected jointly; this treatment, however, while unable to stimulate continuous tumour growth, was able to maintain static growth when plasma PRL was reduced by concurrent injections of $P+P_z+E_g$. From these findings it is postulated that the mechanism of action whereby P maintains static tumour growth is different from that of PRL and independent of circulating PRL levels.**

THE MAJORITY of rat tumours induced by 7,12-dimethylbenz-(a)-anthracene (DMBA) are hormone-dependent, as shown by their regression after ovariectomy (Ox), adrenalectomy (Ax) and hypophysectomy (Pearson *et al.*, 1969). Oestrogens ($E_2$) and prolactin (PRL) have been shown to influence the growth of such tumours (Pearson *et al.*, 1969). The dose–effect relationship of PRL on mammary tumour growth and the duration of PRL-induced stimulation of tumour growth, in the Ax+Ox rat, is a matter of debate. After Ax+Ox, Nagasawa & Yanai (1970) reported that resumption of tumour growth was not only temporary, but stimulated only on high doses of PRL, whilst Pearson *et al.* (1969) found that low or high doses of PRL were equally effective. Reported plasma PRL levels in rats bearing DMBA-induced mammary cancers is also contradictory, both normal (Nagasawa *et al.*, 1973) and raised values having been found (Teller *et al.*, 1977).

Although progesterone (P) enhances induction of DMBA tumours (Jabara, 1967) its role in the growth of established tumours is unclear and conflicting. Horwitz & McGuire (1977) demonstrated that P alone failed to sustain tumour growth after Ax+Ox, despite the initial presence

* Present address: Cumberlnad College of Health Sciences, P.O. Box 170, Lidcombe, N.S.W. 2141, Australia, to which all correspondence should be addressed.

of P receptor (*i.e.*, their results suggested that $E_2$ is an absolute necessity at the tumour site). Kelly *et al.* (1977), on the other hand, reported a similar effect of P, even though the level of $E_2$ was still appreciable, but lowered, by Ox.

The present experiments were designed to clarify the above findings by determining the effects and mechanisms of action of (i) P, (ii) PRL, induced by perphenazine ($P_z$) and (iii) $P + P_z$, on the growth of DMBA-induced mammary carcinomas and on plasma PRL levels in Ax + Ox rats.

### MATERIALS AND METHODS

*Treatment of animals.*—Two hundred and twenty virgin female random-bred Sprague–Dawley rats, weighing $120 \pm 20$ g and fed commercial pellets and tap water *ad libitum*, were housed 5 rats/cage. At 50 days of age, they each received a single intragastric dose of 30 mg DMBA (Eastman Organic Chemicals, U.S.A.) dissolved in 2 ml maize oil. Beginning 4 weeks after DMBA administration, all rats were palpated weekly and any mammary tumour recorded, measured and graphed as described previously (Jabara, 1967). Rats bearing at least one continuously growing tumour were allocated randomly to one of 6 groups (Table I). A tumour was designated as "growing continuously" if the size of the neoplastic mass steadily increased during the course of at least 5 weekly measurements. When these tumours had reached a diameter of ~2 cm, the rats were weighed and given bilateral Ax + Ox. Thereafter they had access to both saline (0·9%) and tap water, and received s.c. injections of deoxycorticosterone acetate (Calbiochem, N.S.W.), 0·1 mg/100 g body weight/2 days, dissolved in 0·1 ml maize oil, to assist sodium retention. When the continuously growing tumours had regressed to half their original preoperative size (14 days on average) (Figs 1–4) the rats were injected s.c., once daily, with oestradiol-17β ($E_2$) (Calbiochem, N.S.W.), progesterone (P) (Sigma Chemical Co., U.S.A.), perphenazine ($P_z$) ("Trilafon", Schering Corp., U.S.A.), ergocornine hydrogenmaleinate ($E_g$) (Sandoz, Switzerland) or combinations, in the doses and for the periods shown in Table I. P and $E_2$ were dissolved in maize oil, $P_z$ in 0·9% saline and $E_g$ in 15% ethanol made up with 0·9% saline. Daily vaginal smears were taken at about 09:00 from all rats in Group 1, to ensure that the animals remained in an oestrus-like state.

Following Ax + Ox, the skin over each tumour was kept shaved and each neoplasm was measured and graphed twice weekly until the end of the experiment. At necropsy each rat was weighed, and portions of each mammary tumour were labelled as to site and side, fixed in 10% buffered formalin and 5μm paraffin sections were stained with haematoxylin and eosin.

*Blood sampling and PRL assay.*—As ether anaesthesia has been shown to increase serum PRL levels in experimental animals (Linke & Niswender, 1972), each rat was etherized for a standard period of 40 sec before ~0·4 ml of blood from the caudal vein was collected into heparinized tubes, just before a particular hormone injection, and at various times between 1 and 30 days afterwards. The blood was centrifuged at 1100 *g* for 10 min, and the plasma stored at −20°C until PRL levels were assayed.

Plasma PRL was measured by the radioimmunoassay method supplied with the NIAMD kit, with only minor modifications. Duplicates were run for each sample and, in order to avoid interassay variation, samples from a complete experiment were assayed at the same time. Results were expressed in terms of the NIAMD-Rat Prolactin-RP-1 standard supplied with the kit.

*Statistical analysis.*—The patterns of growth of the mammary carcinomas were calculated from the slopes for the regression lines for individual rats under similar treatments, by the method described by Rees & Westwood (1974), except that the present data did not require a logarithmic transformation of the results, as they fitted straight lines. Comparison of the growth characteristics of 2 treatment groups was made by comparing the common slope for each group by *t* test. A common slope for the regression lines for rats under similar treatments can be justified by an analysis of variance for all points for all rats under that treatment. A pooled estimate for this slope was used in the calculations (Armitage, 1971).

Comparison of the mean serum prolactin concentrations between different treatments was made by *t* test, the correlation coefficients being computed in the standard manner (Schefler, 1969).

### RESULTS

### *Tumour incidences*

Of 220 rats fed DMBA, 194 (88%) survived the initial toxic effects of the carcinogen and 153 (79%) of these developed mammary tumours after an average latent period of 86 days. Fifty (33%) rats bearing neoplasms showed a continuous growth pattern for at least one of their tumours, and only 40 (79%) of these responded to Ax + Ox by a marked regression in their size, indicating their hormone-dependence. Only these hormone-responsive neoplasms were used in the present experiments.

### *Effects of treatments on survival of rats*

$E_2$, P and $E_g$ treatments administered at the doses stated in Table I did not affect the survival of rats in Groups 1 and 2. The lowest dose of $P_z$ (0·1 mg/100 g/day) also caused no noticeable change in behaviour or appearance of the rats in Groups 3 and 4. Doses higher than this (Groups 5 and 6) caused drowsiness or stupor and weight loss, and only 11% of the rats receiving daily doses of $P_z$ between 0·15 and 0·5 mg/100 g survived up to 10 days. Combined $P + P_z$ administration to rats in Group 5 counteracted the loss in body weight observed with $P_z$ only (0·3 mg/100 g/day) but the sedative effect of $P_z$ appeared to make them more susceptible to infection, resulting in several deaths from pneumonia.

TABLE I.—*Hormone and drug regimes administered to 6 groups of bilaterally adreno-ovariectomized rats bearing partially regressed DMBA-induced mammary carcinomas*

| Group | Treatment | Dose (mg/100 g/day) |
|---|---|---|
| 1 | $E_2$ | 0·0015* |
| | $E_2 + E_g$ | 0·0015* + 0·15 |
| 2 | P | 3* |
| | PX△ | 0 |
| | P | 3* |
| 3 | $P_z$ | 0·1 |
| | $P_z$X△ | 0 |
| 4 | $P + P_z$ | 3* + 0·1 |
| 5 | $P_z$ | 0·3 |
| | $P_z$X△ | 0 |
| | $P_z$ | 0·3 |
| | $P_z + P$ | 0·3 + 3* |
| | $P_z + P + E_g$ | 0·3 + 3* + 0·15 |
| 6 | $P_z$ | 0·15 |
| | $P_z$ | 0·20 |
| | $P_z$ | 0·25 |
| | $P_z$ | 0·30 |
| | $P_z$ | 0·40 |
| | $P_z$ | 0·50 |

* Dose administered as mg/day.
△ "X" indicates cessation of treatment.

### *Effects of hormones and drugs on mammary-cancer growth*

Adreno-ovariectomy caused a marked regression in the size of continuously growing neoplasms ($P < 0·01$). $E_2$ induced reactivation of tumour growth ($P < 0·01$), whereas $E_2 + E_g$ decreased the size of these neoplasms ($P < 0·01$).

Injections of P (3 mg/day for 30 days), in contrast, failed to reactivate tumour growth after Ax + Ox (Fig. 1). The tumours regressed rapidly, however, when P injections were discontinued ($P < 0·01$) and remained static upon resumption of the P regime (Fig. 1). Similarly, daily injections of $P_z$ (0·1 mg/100 g) or combined $P + P_z$ treatment to Groups 3 and 4, respectively, did not reactivate tumour growth (Figs. 2 and 3); the tumours regressed further after withdrawal of the $P_z$ regime in Group 3 ($P < 0·05$) (Fig. 2).

Doses of $P_z > 0·1$ mg/100 g/day increased the tumour growth rate in proportion to the dose (Table II). This correlation was significant ($P < 0·001$) within the limitations of the data, the rats so-treated only surviving between 4 and 10 days (Table II). Of particular interest was the finding that combined $P + P_z$ (0·3 mg/100 g/day) treatment to animals in Group 5 gradually increased their tumour sizes over 21 days, but the addition of $E_g$ to this combination appeared to revert the neoplastic growth pattern to a static one (Fig. 5) for at least 7 days (the rats died at this point).

FIG. 1.—Effects of progesterone (P) on growth of mammary tumours from 3 rats in Group 2 (□) and their mean plasma prolactin levels (±s.d.) (■).

FIG. 2.—Effects of perphenazine ($P_Z$) on growth of mammary tumours from 6 rats in Group 3 (□) and their mean plasma prolactin levels (±s.d.) (■).

FIG. 3.—Effects of progesterone (P) and progesterone + perphenazine ($P + P_Z$) on growth of mammary tumours from 6 rats in Group 4 (□) and their mean plasma prolactin levels (± s.d.) (■).

TABLE II.—*Effects of various perphenazine ($P_Z$) doses on tumour size, plasma prolactin (PRL) levels and survival of bilaterally adreno-ovariectomized (Ax + Ox) DMBA-induced mammary tumour-bearing rats of Group 6*

| Rat No. | $P_Z$ dose (mg/100 g/day) | Tumour size (cm) At Ax + Ox | At start of $P_Z$ treatment* | At death | Increase per day† | Survival (days)† | Plasma PRL (ng/ml ± s.d.)‡ Time after treatment (days) 1 | 7 |
|---|---|---|---|---|---|---|---|---|
| 1 | 0·15 | 1·6 | 0·8 | 1·0 | 0·05 | 4 | 77 ± 2 | |
| 2 | 0·15 | 1·7 | 0·85 | 1·4 | 0·05 | 10 | | 110 ± 7 |
| 3 | 0·20 | 1·6 | 0·8 | 1·4 | 0·06 | 10 | 110 ± 7 | 155 ± 3 |
| 4 | 0·20 | 1·6 | 0·8 | 1·4 | 0·07 | 9 | | |
| 5 | 0·25 | 1·9 | 0·95 | 1·35 | 0·07 | 6 | 153 ± 8 | |
| 6 | 0·25 | 1·7 | 0·85 | 1·35 | 0·07 | 7 | | 215 ± 6 |
| 7 | 0·30 | 2·1 | 1·05 | 1·5 | 0·09 | 5 | 281 ± 5 | |
| 8 | 0·30 | 1·9 | 0·95 | 1·72 | 0·11 | 7 | | 328 ± 11 |
| 9 | 0·40 | 1·6 | 0·8 | 1·2 | 0·10 | 4 | 355 ± 22 | |
| 10 | 0·50 | 2·1 | 1·05 | 2·1 | 0·12 | 8 | | 556 ± 9 |
| 11 | 0·50 | 1·75 | 0·9 | 1·5 | 0·12 | 5 | 463 ± 20 | |

* 15 ± 1 days after Ax + Ox.

† From start of $P_Z$ treatment until death.

‡ PRL was assayed in duplicate.

*Effects of hormone and drug treatments on plasma prolactin levels*

Fifteen days' treatment with $E_2$ increased the plasma prolactin (PRL) concentration ($P < 0{\cdot}001$) above the level in the Ax+Ox control group ($11{\cdot}2 \pm 5{\cdot}4$ ng/ml). Administration of $E_g + E_2$ to these rats significantly depressed the plasma PRL to Ax+Ox levels ($P < 0{\cdot}001$). In contrast, P treatment did not alter the plasma PRL significantly from Ax+Ox levels, irrespective of the duration of its administration (Fig. 1). $P_z$ (0·1 mg/100 g/day) increased the concentrations markedly above Ax+Ox levels ($P < 0{\cdot}05$) (Fig. 2). Cessation of $P_z$ injections led to a significant reduction in PRL level within 24 h ($P < 0{\cdot}001$) decreasing to Ax+Ox levels by 21 days (Fig. 2) Combined $P + P_z$ injections for 24 h to animals in Group 4 significantly increased the PRL levels above those observed when P ($P < 0{\cdot}01$) and $P_z$ ($P < 0{\cdot}01$) were each administered singly for 24 h, and also above Ax+Ox control values ($P < 0{\cdot}01$) (Fig. 3). A gradual but significant rise in the PRL levels was observed after 15 ($P < 0{\cdot}01$) and 30 days ($P < 0{\cdot}01$) respectively after combined $P + P_z$, which were above those obtained at similar intervals after treatment with either hormone alone (Fig. 3).

Increasing the daily dose of $P_z$ to 0·3 mg/100 g significantly raised the plasma PRL levels both above Ax+Ox controls ($P < 0{\cdot}01$) and above that of Group 3 (0·1 mg/100 g/day $P_z$) ($P < 0{\cdot}01$) 24 h after injection (Fig. 4). This value did not alter significantly 24 h after cessation of $P_z$ treatment, but was decreased markedly 15 days later ($P < 0{\cdot}05$). Rats in Group 5 showed significantly raised PRL levels 15 days after resumption of $P_z$ treatment ($P < 0{\cdot}02$). Simultaneous injection of P markedly increased the concentration of PRL, after 10 days ($P < 0{\cdot}05$) and 21 days ($P < 0{\cdot}01$) respectively, when compared with the values

FIG. 4.—Effects of perphenazine ($P_z$), $P + P_z$ and $P + P_z$+ergocornine ($E_g$) on growth of mammary tumours from 2 rats in Group 5 (□) and their mean plasma prolactin levels (±s.d.) (■).

before P treatment; however, the mean increase was only 12% compared with 70% in Group 4. Administration of $E_g$ to these animals, in addition to P and $P_z$, effectively reduced the PRL levels after 24 h ($P < 0{\cdot}01$) and, after 7 days, levels had returned to Ax + Ox control values (Fig. 4).

Daily $P_z$ doses of 0·15–0·5 mg/100 g/day (Table II) all increased the plasma PRL values in Group 6 above those of the Ax + Ox controls within 24 h ($P < 0{\cdot}05$). PRL titres were significantly increased in proportion to the dose of $P_z$ ($P < 0{\cdot}05$ in each case, except for 0·4 mg/100 g/day $P_z$, which could not be calculated since only one rat survived).

### *Correlation between tumour size and plasma PRL*

A significant correlation was shown between plasma PRL levels and corresponding tumour sizes (Figs. 1–4) in Groups 1 and 2 and in those animals in Group 6 which received daily treatments with 0·15–0·3 and 0·5 mg/100 g $P_z$ ($P < 0{\cdot}05$); there also appeared to be a correlation between tumour size and PRL value in rats treated with 0·4 mg/100 g/day $P_z$, but statistical analysis was not possible as only one animal survived this regime for long enough. No significant correlations between PRL levels and tumour sizes were found in Groups 3 and 4, nor in Group 5, after a combination of $P + P_z + E_g$. However, a correlation was apparent in Group 3 when $P_z$ administration was discontinued (Fig. 2) and in Group 5 when only $P + P_z$ was given (Fig. 4).

### *Tumour types*

All neoplasms used in these experiments were carcinomas. Tumours from animals in all groups other than Group 5, weighed up to 3 g and measured up to 1 cm (mean diameter); neoplasms from rats in Group 5 weighed up to 25 g and measured up to 3·4 cm (mean diameter). Microscopically, most mammary neoplasms in all 6 groups were papillary cystadenocarcinomas, while a few were classified as adenocarcinomas or solid, poorly differentiated carcinomas (Jabara, 1967). No direct correlation was evident between tumour growth behaviour and histology of the carcinomas in Groups 1–6 and, apart from marked degenerative changes in carcinomas after $E_2 + E_g$ (Group 1), there was no apparent relationship between tumour histology and treatment of the host in any of the 6 groups of animals.

### DISCUSSION

In agreement with previous findings (Pearson *et al.*, 1969) $E_2$ administration caused a significant increase in plasma PRL, and also reactivated growth of tumours which had regressed after Ax + Ox. Subsequent concurrent injections of $E_2 + E_g$ induced macroscopic regression of these $E_2$-dependent tumours, gross degenerative histological changes in the neoplasms, and markedly reduced plasma PRL levels (Shaar & Clemens, 1972).

The observation that $P_z$ produced a dose-related increase in plasma PRL confirms the findings of Bogden *et al.* (1974). Perphenazine is thought to raise plasma PRL levels by inhibiting dopaminergic transmission, either through suppression of hypothalamic PRL-inhibitory factor, or antagonism of that factor at the level of the pituitary, or both (Frantz, 1978). However, it is interesting to note that in the present experiment no increase in growth of otherwise static, hormone-responsive tumours was apparent unless $P_z$ was administered in doses of 0·15 mg/100 g/day or more. In other words, a low dose of prolactin (*i.e.* 0·1 mg/100 g/day) was insufficient to reactivate tumour growth, but was able to maintain static growth by slightly stimulating the tumour in order to sustain growth in the static phase. Active growth, on the other hand, appeared to require a certain minimal level of plasma PRL, as was also suggested from the work of Nagasawa & Yanai (1970). They demonstrated that injections of ovine PRL (1·25 mg, twice daily for 20

days) to Ax + Ox rats bearing DMBA-induced mammary carcinomas, reactivated growth of the tumours only for the first 10 days, after which the neoplasms regressed; injection of the same dose of PRL 20 days after Ax + Ox had no effect on tumour growth. Enhancement of both the level of plasma PRL and of tumour growth up to 14 days from the start of injections did not occur until higher doses of $P_Z$ (0·3 mg/100 g/day) were administered in accord with the findings of Nagasawa & Yanai (1970). However, the duration of PRL-induced stimulation of tumour growth did not appear to be limited. Whether the tumours became so large as to render growth autonomous (Griswald & Green, 1970; Huggins & Yang, 1962) or whether growth simply cannot be sustained (Nagasawa & Yanai, 1970; Klaiber *et al.*, 1969) beyond 14 days needs to be studied further, especially the effects of $P_Z$ at doses > 0·15 mg/100 g/day and for longer periods. Unfortunately, this was not possible in the present experiments because, in contrast to the findings of Pearson *et al.* (1969), high mortality was observed when $P_Z$ doses exceeded 0·1 mg/100 g/day; this may be due to the substrain of Sprague–Dawley rat used in the present experiments being more susceptible to this drug.

The present findings, as well as those of Nagasawa & Yanai (1970), are in direct contrast to reports by Sinha *et al.* (1973) which suggest that PRL alone, in the absence of $E_2$, is insufficient to maintain growth of DMBA-induced tumours, ovarian hormones being necessary to maintain the sensitivity of the tumour to the PRL effect. The present results may be explained if prolactin is assumed to amplify its own receptor levels in the mammary gland, as suggested by Djiane & Durand (1977). Furthermore, breast cancers contain a heterogeneous cell population within a single tumour, and among different tumours, and it is conceivable that the rate of tumour growth in response to a particular hormone (*e.g.* PRL) might depend on the rate of cell division of PRL-dependent cells within it. It may be postulated, therefore, that the high dose of $P_Z$ (0·3 mg/100 g/day) would increase the level of plasma PRL and the number of PRL receptors, and hence cause a far greater increase in the rate of division of existing PRL-dependent cells than would a low dose of $P_Z$ (0·1 mg/100 g/day) which only maintained static tumour growth (*i.e.* a growth pattern which requires an equilibrium between cell gain and cell loss).

Progesterone alone, though without effect on PRL levels, was found to maintain static tumour growth (*i.e.* it exerted a slight stimulatory effect on the tumour to sustain growth in a static phase). Kim (1965) also reported a similar effect of P on 3-methylcholanthrene-induced mammary tumours. P may possibly have some direct metabolic effect on the growth of these tumours, growth *per se* not appearing to be the important factor, but rather the biochemical consequence(s) of P. In any event it is interesting to note that P acts independently of circulating plasma PRL. Therefore the present findings indicate that P apparently acts by a different mechanism from PRL to maintain static growth, especially in view of the fact that $P + P_Z$ high-dose treatment caused an additive increase in circulating PRL levels. It is postulated that P may be acting directly at the tumour site (Asselin *et al.*, 1976) by inducing the synthesis of sufficient P receptor to maintain, but not increase, tumour growth. More extensive studies are needed, especially in the field of P receptors and the mapping of the complex sequence of events which accompany receptors, to evaluate this concept further. However, it seems unlikely that P is acting *via* an effect on PRL secretion since, despite basal Ax + Ox control PRL levels recorded after P and $P + P_Z + E_g$ treatments (Groups 2 and 5, respectively), tumour growth remained static and did not regress as would have been expected if PRL were involved. In addition, the hypothesis that $E_2$ is mandatory for tumour growth cannot be supported by the present data, because 14 days after Ax + Ox, when plasma $E_2$ levels would be expected to be minimal, it was noted that (i) high doses of $P_z$ were effective in markedly increasing tumour growth, and (ii) tumour growth remained static after P and $P + P_z + E_g$ treatments. The first observation of this series is in agreement with that of Leung *et al.* (1975), who found that PRL stimulated the growth of some endocrine-ablation-responsive tumours 7 or 11 days after Ax + Ox, but required $E_2$ as well to stimulate others. The second observation contrasts with the report by Horwitz & McGuire (1977) and even more so with that of Kelly *et al.* (1977), whose finding that P cannot maintain tumour growth after Ox in the presence of low but appreciable levels of plasma $E_2$ is puzzling. Furthermore, the fact that rats receiving 3 mg P daily remain in almost continuous dioestrus (Jabara *et al.*, 1972) fails to confirm the suggestion of Baggett *et al.* (1956) that P may be converted to $E_2$ *in vivo*.

This study does not negate the importance of PRL and $E_2$ in the growth of DMBA-induced mammary neoplasms, but suggests that alongside these hormones P may also play a vital part in the promotional stage of mammary carcinogenesis.

The kit for determination of plasma prolactin levels was generously provided through the National Institute of Arthritis and Metabolic Diseases (NIAMD) National Institutes of Health Rat Pituitary Hormone Distribution Program.

The authors wish to thank Dr J. D. Wingfield, Essex Laboratories Pty Ltd, N.S.W., for donating the perphenazine, and Dr M. E. Wilkins, Sandoz Australia Pty Ltd, N.S.W., for donating the ergocornine hydrogenmaleinate. The assistance of Mr R. J. Rowlands, Division of Protein Chemistry, CSIRO, Melbourne, with the statistical analysis is also gratefully acknowledged.

This work was carried out during the tenure of a grant from the Anti-Cancer Council of Victoria to one of us (A.G.J.).

### REFERENCES

ARMITAGE, P. (1971) Regression and correlation. In *Statistical Methods in Medical Research*. Oxford: Blackwell Scientific Publications. p. 147.

ASSELIN, J., LABRIE, F., KELLY, P. A., PHILIBERT, D. & RAYNAUD, J. P. (1976) Specific progesterone receptors in dimethylbenzanthracene (DMBA)-induced mammary tumors. *Steroids*, **27**, 395.

BAGGETT, B., ENGEL, L. L., SAVARD, K. & DORFMAN, R. I. (1956) The conversion of testosterone-3-$C^{14}$ to $C^{14}$-estradiol-17 by human ovarian tissue. *J. Biol. Chem.*, **221**, 931.

BOGDEN, A. E., TAYLOR, D. J., KUO, E. Y. H., MASON, M. M. & SPEROPOULOS, A. (1974) The effect of perphenazine-induced serum prolactin response on estrogen-primed mammary tumor-host systems, 13762 and R-35 mammary adenocarcinomas. *Cancer Res.*, **34**, 3018.

DJIANE, J. & DURAND, P. (1977) Prolactin–progesterone antagonism in self regulation of prolactin receptors in the mammary gland. *Nature*, **266**, 641.

FRANTZ, A. G. (1978) Prolactin. *N. Engl. J. Med.*, **298**, 201.

GRISWALD, D. P., JR & GREEN, C. H. (1970) Observations on the hormone sensitivity of 7,12-dimethylbenz(a)anthracene-induced mammary tumors in the Sprague–Dawley rat. *Cancer Res.*, **30**, 819.

HORWITZ, K. B. & MCGUIRE, W. L. (1977) Progesterone and progesterone receptors in experimental breast cancer. *Cancer Res.*, **37**, 1733.

HUGGINS, C. & YANG, N. C. (1962) Induction and extinction of mammary cancer. *Science*, **137**, 257.

JABARA, A. G. (1967) Effects of progesterone on 9,10-dimethyl-1,2-benzanthracene-induced mammary tumours in Sprague–Dawley rats. *Br. J. Cancer*, **21**, 418.

JABARA, A. G., TOYNE, P. H. & FISHER, R. J. (1972) An autoradiographic study of the early effects of 7,12-dimethylbenz(a)anthracene and progesterone on DNA synthesis in rat mammary epithelial cells and subsequent tumour development. *Br. J. Cancer*, **26**, 265.

KELLY, P. A., ASSELIN, J., LABRIE, F. & RAYNAUD, J. P. (1977) Regulation of hormone receptor levels and growth of DMBA-induced mammary tumors by RU16117 and other steroids in the rat. In *Progesterone Receptors in Normal and Neoplastic Tissues*. Ed. McGuire *et al.* New York: Raven Press. p. 85.

KIM, U. (1965) Pituitary function and hormonal therapy of experimental breast cancer. *Cancer Res.*, **25**, 1146.

KLAIBER, M. S., GRUENSTEIN, M., MERANZE, D. R. & SHIMKIN, M. B. (1969) Influence of hypothalamic lesions on the induction and growth of mammary cancers in Sprague–Dawley rats receiving 7,12-dimethylbenz(a)anthracene. *Cancer Res.*, **29**, 999.

LEUNG, B. S., SASAKI, G. H. & LEUNG, J. S. (1975) Estrogen-prolactin dependency in 7,12-dimethylbenz(a)anthracene-induced tumors. *Cancer Res.*, **35**, 621.

LINKE, D. M. & NISWENDER, G. D. (1972) Serum levels of prolactin luteinizing hormone and follicle-stimulating hormone during pregnancy in the rat. *Endocrinology*, **90**, 632.

NAGASAWA, H., CHEN, C. L. & MEITES, J. (1973) Relation between growth of carcinogen-induced mammary cancers and serum prolactin values in rats. *Proc. Soc. Exp. Biol. Med.*, **142**, 625.

NAGASAWA, H. & YANAI, R. (1970) Effects of prolactin or growth hormone on growth of carcinogen-induced mammary tumours of adreno-ovariectomized rats. *Int. J. Cancer*, **6**, 488.

PEARSON, O. H., LLERENA, O., LLERENA, L., MOLINA, A. & BUTLER, T. (1969) Prolactin-dependent rat mammary cancer: A model for man? *Trans. Ass. Am. Physicians*, **82**, 225.

REES, J. A. & WESTWOOD, M. (1974) A method of comparing differences in tumour growth rates applied to a study of the increasing growth capacity of mouse carcinomata. *Br. J. Cancer*, **29**, 151.

SCHEFLER, W. C. (1969) Correlation coefficients. In *Statistics for the Biological Sciences*. Massachusetts: Addison-Wesley. p. 140.

SHAAR, C. J. & CLEMENS, J. A. (1972) Inhibition of lactation and prolactin secretion in rats by ergot alkaloids. *Endocrinology*, **90**, 285.

SINHA, D., COOPER, D. & DAO, T. L. (1973) The nature of estrogen and prolactin effects on mammary tumorigenesis. *Cancer Res.*, **33**, 411.

TELLER, M. N., STOCK, C. C., HELLMAN, L. & 5 others (1977) Comparative effects of a series of prolactin inhibitors, 17$\beta$-estradiol and 2$\alpha$-methyldihydrotestosterone propionate, on growth of 7,12-dimethylbenz(a)anthracene-induced rat mammary carcinomas. *Cancer Res.*, **37**, 3932.